# Vitreous Hemorrhage as Presenting Sign of Retinal Arteriovenous Malformation

**DOI:** 10.1155/2020/8858242

**Published:** 2020-10-19

**Authors:** Geraldine P. B. M. Accou, Fanny Nerinckx, Bart P. Leroy, Julie De Zaeytijd

**Affiliations:** ^1^Department of Ophthalmology, Ghent University Hospital, Ghent, Belgium; ^2^Department of Ophthalmology & Center for Medical Genetics, Ghent University Hospital, Ghent, Belgium; ^3^Children's Hospital of Philadelphia, Philadelphia, PA, USA

## Abstract

**Objective:**

To describe a patient with vitreous hemorrhage and peripheral retinal ischemia, eventually diagnosed with an underlying retinal arteriovenous malformation.

**Methods:**

A 15-year-old girl presented with sudden-onset, painless visual loss in the right eye. She underwent a full ophthalmological work-up.

**Results:**

BCVA was less than 20/400 in the right eye and 20/20 in the left eye. Intraocular pressure and anterior segment examination were unremarkable. Fundoscopy was impossible due to an opaque vitreous hemorrhage in the right eye. The left eye was completely unremarkable. Examination during a 23-gauge pars plana vitrectomy showed dilated, tortuous arteriovenous vessels extending from the optic disc and silver wiring of the enlarged vessels. A clinical diagnosis of retinal arteriovenous malformation was made. During surgery, a peripheral retinal photocoagulation was executed to avoid rebleeding. Postoperatively, fluorescein angiography demonstrated additional macular microangiopathy and diffuse retinal nonperfusion in the periphery. The MRI brain revealed neither cerebral nor orbital vascular anomaly, confirming a group 2 retinal arteriovenous malformation.

**Conclusion:**

Retinal arteriovenous malformations are generally considered stable over time. However, complications due to retinal ischemia can occur. Hence, regular observation is warranted. In so doing, timely treatment can be offered to avoid complications.

## 1. Introduction

Retinal arteriovenous malformation is a rare, unilateral sporadic disorder. It is characterized by anomalous arteriovenous malformations of the retina, looking like tangle-like loops of small arteries entering into retinal veins. The malformations can be both local or extensive and can be distributed over the entire retina [1]. If local, they are predominantly found either close to the optic disc or in the temporal retinal periphery. If associated with cerebral arteriovenous malformations and facial nevi, the condition is known as the Wyburn-Mason syndrome [2, 3].

In 1973, Archer et al. published a classification of retinal arteriovenous malformations, which is still in use today. In short, group 1 is characterized by small lesions with an abnormal capillary plexus in between, group 2 by arteriovenous malformations without intervening capillary bed, and group 3 by extensive malformations where no distinction can be made between arteries and veins [3].

Retinal arteriovenous malformation can cause vision loss via various ocular complications [4, 5–7]. This case report presents a young patient in whom the AV malformation had been previously undiagnosed, who presented with a vitreous hemorrhage.

## 2. Case Description

A 15-year-old girl presented with a history of sudden-onset, painless visual loss in the right eye, since 3 months. Generally, she was in good health, and her family history was unremarkable. The best-corrected visual acuity was less than 20/400 in the right eye and 20/20 in the left eye. Intraocular pressure and slit-lamp examination were unremarkable in both eyes. Fundoscopy was impossible due to a dense vitreous hemorrhage blocking the view of the fundus in the right eye. Ocular ultrasound confirmed the vitreous hemorrhage in the absence of a retinal detachment, a retinal tear, or an intraocular mass. A preoperative etiological work-up including fundoscopy and fluorescein angiography of the left eye to identify common retinal vascular or inflammatory pathologies was negative.

**Figure 1:**
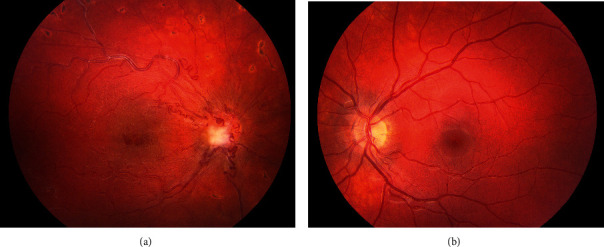
(a) Fundus of the affected right eye of patient postsurgery, showing enlarged and tortuous vessels extending from the optic disc. A fibrous tuft is visible at the optic disc. (b) Normal fundus of the left eye.

**Figure 2:**
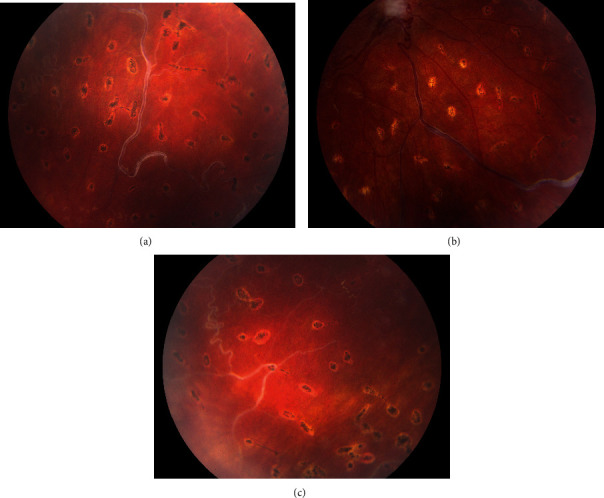
Silver wiring of the retinal veins in the superior (a), inferior (b), and temporal quadrant of the retina (c).

Without spontaneous clearing, the vitreous hemorrhage of the right eye was treated with a 23-gauge pars plana vitrectomy, which revealed dilated and tortuous retinal arteries as well as veins, extending from the optic disc with multiple communications and with some of them crossing the midline (Figure 1). There was an intraretinal hemorrhage in the temporal retinal periphery, but no exudations. Three neovascular tufts were visible in the inferior retinal periphery. Silver wiring of the retinal veins was pronounced in all four quadrants of the retina (Figure 2). Because of silver wiring, peripheral retinal ischemia was suspected. The combination of the retinal vascular malformation on the optic disc, with neovascular tufts in the inferior retinal periphery, led to a diagnosis of vitreous hemorrhage with neovascularization. An intravitreal injection of bevacizumab was given during surgery to avoid rebleeding. A peripheral pan-retinal photocoagulation was performed to aim for long-term stabilization.

**Figure 3:**
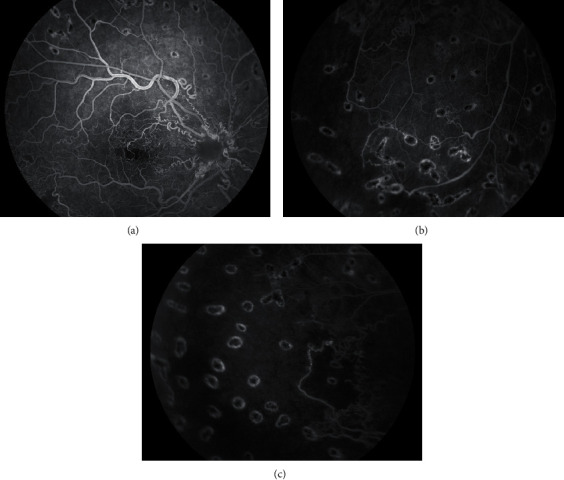
Fluorescein angiography of the affected right eye of the patient. (a) Abnormal arteriovenous communications with microangiopathy in the macula and an enlarged foveal avascular zone. (b, c) Diffuse capillary drop out in retinal periphery. No peripheral neovascularization is observed; however, pictures were taken 2 weeks after peroperative intravitreal anti-VEGF injection.

Two weeks postsurgery, the BCVA in the right eye had improved to 20/60. Fluorescein angiography of the right eye demonstrated abnormal arteriovenous communications with an altered capillary network in the macula and diffuse ischemia in the retinal periphery (Figure 3). No peripheral retinal neovascularization was observed, possibly because of the intravitreal anti-VEGF injection administered during surgery.

Based on the above, a diagnosis of arteriovenous malformation group 2 (classification by Archer et al.) was made. Additional magnetic resonance imaging (MRI) of the brain revealed no abnormality.

## 3. Discussion

Arteriovenous malformations can remain stable over many years and are therefore usually thought of as benign and stationary lesions [8]. However, during follow-up, retinal complications cannot be considered rare, with a reported prevalence as high as 24.6%. Retinal hemorrhages are the most frequent (28.1%), followed by retinal vein occlusion (17.5%) and vitreous hemorrhage (10.5%). Evolution towards neovascular glaucoma on the other hand is rare (0.05%) [1, 9–11, 12].

Arteriovenous malformations are high-flow systems because of direct communication between retinal arteries and veins, with either an abnormal or completely absent interposing capillary plexus. Consequently, the hydrostatic pressure in the retinal veins is a lot higher than normal and exposes the veins to arterial blood pressure levels. Considering a majority of the complications imply ischemia related etiology, three hypothetical mechanisms have been put forward to explain the mechanism of the underlying retinal ischemia. The first mechanism states that this abnormally elevated hydrostatic venous pressure causes endothelial injury of the vessel wall, leading to thrombosis. The second hypothesis discusses a “steal phenomenon.” Due to the increased venous flow in the absence of a capillary network, the retinal perfusion is decreased, leading to retinal and/or choroidal ischemia. The third mechanism states direct compression of the veins due to the arteriovenous malformation at arteriovenous crossing sites, the more common mechanism for retinal vein occlusion [1, 9, 11, 13, 14].

In the patient described in this report, the mechanism behind the vitreous hemorrhage is most likely a combination of the elevated hydrostatic pressure, given the amount of silver wiring vessels, and the steal phenomenon, leading to peripheral retinal ischemia. Indeed, the lack of numerous intraretinal hemorrhages and the absence of filling defects on fluorescein angiography argue against damage to the vessel wall with subsequent occlusion.

The ocular management of retinal arteriovenous malformations is observation of the lesions or, if required, treatment of the ocular complications. Gass already emphasized that relatively minor alterations of retinal vessels may occur in association with central nervous system vascular anomalies [15]. Thus, whenever confronted with a retinal arteriovenous malformation, the neurologic evaluation with MRI imaging of the brain is always indicated.

## 4. Conclusions

The etiology of a vitreous hemorrhage varies according to the characteristics of the patient population. In patients younger than 40 years, the most frequent cause is an ocular trauma. If there is no evidence of a trauma, a work-up, including laboratory testing and fluorescein angiography is indicated. If the etiology is unclear after work-up, and no visualization of the retina is possible, pars plana vitrectomy is indicated.

A retinal arteriovenous malformation is part of the differential diagnosis of a vitreous hemorrhage in an otherwise healthy person. Although retinal arteriovenous malformations are thought of to be stable over time, complications, especially due to retinal ischemia, might occur. Observation, in combination with fluorescein angiography, is warranted. As such, timely treatment can be offered to avoid complications.
